# SOFIE: Surgery for Olecranon Fractures in the Elderly: a randomised controlled trial of operative versus non-operative treatment

**DOI:** 10.1186/s12891-015-0789-6

**Published:** 2015-10-27

**Authors:** Michael Symes, Ian A. Harris, John Limbers, Mithun Joshi

**Affiliations:** Gosford Hospital, Holden St, Gosford, NSW 2250 Australia; Whitlam Orthopaedic Research Centre, Ingham Institute for Applied Medical Research, and South Western Sydney Clinical School, UNSW Australia, 1 Campbell St, Liverpool, NSW 2170 Australia

**Keywords:** Olecranon, Fracture, Randomised controlled trial, Non-operative, Operative, Open reduction, Internal fixation, Geriatric

## Abstract

**Background:**

Displaced olecranon fractures after a simple fall are common in elderly patients. This patient group often has multiple medical co-morbidities and low functional demands. Standard treatment for these fractures has been operative fixation, using either wires or a plate. Recent case series suggest that such injuries can be managed without surgery with good functional outcomes. There has been no published trial comparing operative to non-operative treatment for displaced olecranon fractures.

This project aims to test for superiority of operative treatment versus non-operative treatment for displaced olecranon fractures in the elderly, by comparing pain and function in the affected limb up to one year after the injury.

**Methods/Design:**

SOFIE is an international study with a multicentre pragmatic randomised controlled trial design. The primary objective of the study is to compare a patient related outcome, the Disability of the Arm Shoulder and Hand (DASH) Score, between patients treated operatively and non-operatively at twelve months.

Patients will be considered for the study if they are 75 years of age or older, medically fit for surgery, have an isolated displaced olecranon fracture, and present within 14 days of injury. Eligible patients willing to participate will be randomised either to operative fixation, with surgery using the preferred technique of the treating orthopaedic surgeon (tension band wiring or plate fixation), or to non-operative treatment involving early range of motion as tolerated.

Secondary outcome measures will include pain, active range of motion, elbow extension strength, and any adverse events (infection, secondary interventions) at 3 and 12 months.

**Discussion:**

The study will answer an important clinical question about the effectiveness of a commonly performed orthopaedic procedure, and will guide future treatment for displaced olecranon fractures in the elderly.

**Trial registration number:**

World Health Organisation Universal Trial Number (WHO UTN) - U111111574090.

Australian and New Zealand Clinical Trials Registry (ANZCTR) - ACTRN12614000588695.

**Electronic supplementary material:**

The online version of this article (doi:10.1186/s12891-015-0789-6) contains supplementary material, which is available to authorized users.

## Background

The incidence of olecranon fractures in the adult population is 11.5 per 100,000 per year. These fractures make up 20 % of all proximal forearm fractures, and 8–10 % of all elbow fractures. Recent studies have reported an increasing incidence of olecranon fractures in the elderly population [1, 2]. In this age group, they are predominantly fragility fractures seen in patients with multiple co-morbidities and low functional demands [3, 4].

The current standard treatment for displaced olecranon fractures, based on case series and tradition, is operative fixation with either tension band wiring or plate fixation [5–10]. Despite being commonly performed, the results from studies investigating outcomes and complications after operative fixation are conflicting, especially in the elderly with osteoporotic bone. Reported complications include those related to anaesthesia, as well as those specific to the surgical fixation (wire migration, soft- tissue irritation, olecranon bursitis, wire breakage, fracture displacement and the need for a second operation for removal of hardware, with rates as high as 70 %) [11–16].

Further to this high complication rate, there is recent evidence from case series that non-operative management of displaced olecranon fractures is a reasonable option in elderly patients, with comparable outcomes without the risk of anaesthetic and surgical complications [10, 17–19].

Despite extensive case series data, we are unaware of any study directly comparing operative and non-operative management of displaced olecranon fractures in the elderly. The aim of our study is to perform a randomized control trial testing for superiority of operative treatment over non-operative treatment in restoring upper limb function for displaced olecranon fractures in the elderly.

## Methods and design

**Fig. 1:**
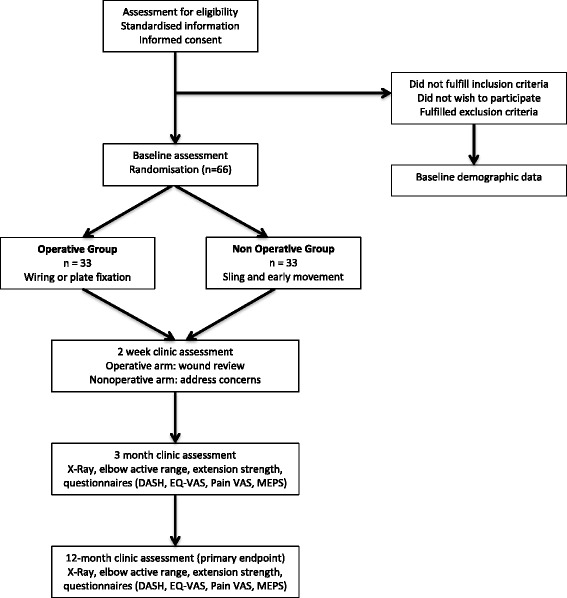
Patient flow through study

SOFIE is a prospective multicentre trial conducted at sites in Australia and New Zealand. The study uses a pragmatic randomised controlled trial design. The study flowchart is provided in Fig. 1.

### Ethics

SOFIE has been approved by the Hunter New England Research Ethics and Governance Unit for all recruiting sites. (HNEHREC Reference No: 13/10/16/4.04, NSW HREC Reference No: HREC/13/HNE/405, These recruiting sites in alphabetical order are Cairns Base Hospital; Concord General Repatriation Hospital; Dubbo Base Hospital; Frankston Hospital; Gosford Hospital; John Hunter Hospital; Liverpool Hospital; Mackay Base Hospital; Nambour General Hospital; Nepean Hospital; Orange Base Hospital; Prince of Wales Hospital; Royal Adelaide Hospital; Royal Brisbane Hospital; Royal North Shore Hospital; Royal Prince Alfred Hospital; Ryde Hospital; St George Hospital; Sutherland Hospital; Wellington Hospital and Wyong Hospital.

The study was registered on the 03/06/2014 with both the Australian and New Zealand Clinical Trials Registry (ACTRN12614000588695) and the World Health Organisation Universal Trial Registry (WHO UTN - U111111574090).

### Study population

Patients will be considered for the study if they are 75 years or older, medically fit for surgery, have an isolated displaced (more than 2 mm) olecranon fracture based on standard anteroposterior (AP) and lateral radiographs, and present within 14 days of injury to a participating institution.

Patients will be excluded if they are unable to provide consent due to limited English proficiency or cognitive impairment, have other fractures about the elbow (coronoid, radial head, other ulna or distal humerus fractures), an associated ligamentous injury, a subluxed or dislocated elbow, a flipped fragment (proximal fragment rotates 180° so that the rough trabecular surface of the fracture is in direct contact with overlying skin which can cause skin breakdown), or have an open injury.

### Recruitment

Recruitment of patients who meet study eligibility criteria will involve an explanation of the rationale for the study. This will be supplemented with a patient information sheet (see Additional file 1). Patients willing to participate will be asked to sign a consent form. Enrolled patients will then be randomised to either operative or non-operative management by using a central web-based randomisation service. Basic demographic data will be collected for both groups to look at independent variables including age, gender, pre-injury difficulty using arm, comminution (yes/no), diabetes (yes/no), smoking status (current smoker: yes/no) and operation type (wiring or plating) (see Additional file 2). Limited de-identified demographic data (age, gender, hand dominance, residential status) will also be collected on patients who choose not to participate, or are ineligible to participate due to inability to consent. This information will be used to determine selection bias.

### Randomisation

Consented patients will be randomised to either operative or non-operative management by using a central web-based randomisation service. Randomisation will be stratified by site.

### Interventions

The operative group will undergo surgical fixation by the preferred technique of the treating surgeon (tension band wiring or plate fixation) in a manner consistent with the usual care of the participating institution. The post operative regime will include immobilisation in a long-arm backslab for 2 weeks followed by progressive range of motion as tolerated. This pragmatic approach to surgical intervention more accurately reflects current practice and improves study feasibility and acceptance, and generalisability of the results.

Non-operative treatment will consist of a sling as needed and immediate progressive range of motion as tolerated. A long-arm plaster splint may be applied for the initial 2 weeks if needed for pain control, followed by movement as tolerated. Any decision on referral for physiotherapy will be left to the treating team for both treatment groups.

Study investigators and participants will not be blinded to treatment group. Blinded researchers, however, will collect the primary outcome measure (DASH score at 12 months) via telephone. The statistician will also be blinded to treatment group.

### Outcome measures

Participants will be followed up at two weeks, three months and twelve months post intervention. The primary outcome will be the Disability of the Arm Shoulder and Hand (DASH) Score at 12 months [20]. The DASH score is a widely used, validated outcome tool used in studies of upper limb injuries and surgery, and is a continuous score on a scale from 0 to 100 [21]. Secondary outcome measures collected at 3 months and 12 months will include DASH, EuroQol Visual Analogue Score (EQ VAS), Pain Visual Analgoue Scale (VAS), Mayo Elbow Performance Score (MEPS), complications, elbow active range of motion, and elbow extension strength.

### Sample size calculation

The study will recruit a total of 65 patients with a minimum of 26 in each group at 12 months. This is based on having 95 % power to detect the minimum clinically important difference (15 points) on the DASH score at a significance level of 0.05 and it also allows for 20 % loss to follow up [8, 9, 21].

### Statistical analysis

A two-sided t test will be used to compare the two independent groups. Adjustment for other variables will not be necessary, as the technique of minimisation will be used during randomisation, and randomisation will be stratified by site. Intention to treat analysis will be performed in the primary analysis. As-treated analysis will be added as a secondary analysis. There will be no interim analysis due to the low risk of adverse events compared to usual care and adverse events will be monitored and reported to the administering institution and project manager. De-identified demographic information collected from ineligible or non-consenting patients will be used to understand selection bias in the study.

### Monitoring and quality assurance

The adverse events to be recorded are infection (any diagnosis of infection resulting in any treatment); re-operation (including reason for reoperation); death (including cause and likelihood of any casual link between treatment or study participation and death) and implant failure (fracture or migration of implants).

Data will be collected by local site investigators and submitted securely to the project manager at the administering institution. Data will be stored in locked filing cabinets and password protected computers within the administering institution. The coordinating and principal investigators are responsible for the adherence to all ethical committee rules and guidelines for the accuracy and completeness of all forms, entries and informed consent.

## Discussion

There are no prospective randomised controlled trials comparing operative and non operative treatment for displaced olecranon fractures in the elderly, with current management based on case series and tradition alone. The SOFIE trial will compare the management of these fractures and in addition to examining a previously unanswered clinical question about the effectiveness of a commonly performed orthopaedic procedure, this study will guide future treatment for this common injury, potentially changing current practice.

## Additional files

Additional file 1:
**SOFIE: Surgery for Olecranon Fractures In the Elderly: a randomised controlled trial of operative versus non-operative treatment.** (DOC 49 kb) Additional file 2:
**Patient Information Sheet.** (DOCX 90 kb)

## Abbreviations

AP: anteroposterior
DASH: disability of the arm shoulder and hand score
EQ VAS: EuroQol visual analogue scale
VAS: visual analogue scale
MEPS: Mayo elbow performance score
