# Supplementary material for: SOFIE: Surgery for Olecranon Fractures in the Elderly: a randomised controlled trial of operative versus non-operative treatment
Source: BMC Musculoskelet Disord. 2015 Oct 27;16:324. doi: 10.1186/s12891-015-0789-6 (PMC4624605; doi:10.1186/s12891-015-0789-6)
Supplement: Additional file 1: — SOFIE: Surgery for Olecranon Fractures In the Elderly: a randomised controlled trial of operative versus non-operative treatment. (DOC 49 kb) [file 12891_2015_789_MOESM1_ESM.doc]

**SOFIE: Surgery for Olecranon Fractures In the Elderly: a randomised controlled trial of operative versus non-operative treatment.**

PARTICIPANT INFORMATION SHEET

You are invited to participate in a research study comparing outcomes between surgical and non-surgical treatment of olecranon (tip of elbow) fractures, similar to the one you have sustained.

Treatment of these fractures is controversial with no current consensus among surgeons on what is best for the patient. Most patients with olecranon fractures are treated with surgery to realign the bones and insert wires or screws to hold the fracture in place. There is, however, recent evidence showing equally good outcomes in patients with these fractures managed without an operation.

The study is being conducted by a group of researchers throughout Australia. Your local researchers are *[Insert name(s) of site-specific researchers]*

Before you decide whether or not you wish to participate in this study, it is important for you to understand why the research is being done and what it will involve. Please take the time to read the following information carefully and discuss it with others if you wish.

1. **What is the purpose of this study?**

By allocating patients to either surgery or non-operative treatment and comparing the outcomes of treatment in both groups, we aim to be able to determine whether there is any advantage in surgery for olecranon fractures for relief of pain, resotration of function, and complications.

**2. Why have I been invited to participate in this study?**

For this study to be suitable for you, you must

1. Be medically fit for an operation
2. Be at least 75 years of age
3. Have an elbow fracture similar to yours
4. Be seen by us within 2 weeks of sustaining the injury

**3. What if I don’t want to take part in this study, or if I want to withdraw later?**

Participation in this study is voluntary. It is completely up to you whether or not you participate. If you decide not to participate, it will not affect the treatment you receive now or in the future. Whatever your decision, it will not affect your relationship with the staff caring for you.

If you wish to withdraw from the study once it has started, you can do so at any time without having to give a reason. If you do decide to withdraw from the study all the information about you collected for the study will be destroyed.

**4. What are the alternatives to participating in this study?**

If you decide not to participate in this study, you will be treated according to usual practice, by your orthopaedic team.

**5. What does this study involve?**

If you agree to participate in this study, you will be asked to sign the Participant Consent Form. You will be allocated to 1 of 2 possible groups: surgery or no surgery. This will be decided using a random allocation computer-based program. You or your doctor cannot determine what group you will be in. You will have a 50% chance of getting either treatment.

If you are in the surgery group, you will have an operation for the elbow fracture. The surgeon looking after you will decide on which operation is best after looking at the nature of your fracture. After the operation, your elbow may be splinted for 2 weeks and you may be asked to see a physiotherapist to assist your recovery.

If you are assigned to the no surgery group, your arm will be placed in a sling or a temporary splint. You may be referred to physiotherapy to assist your recovery.

Regardless of whether you have surgery or not, we will check your elbow at regular intervals (2 weeks, 3 months, and 1 year after the injury or surgery) to check your progress and answer any questions you may have. Each check up will involve some or all of the following:

1. Completion of a questionnaire about your elbow pain and function, any complications, and about your helath in general
2. Measurement of elbow movement and strength
3. X-ray of the affected elbow

**6. Are there risks to me in taking part in this study?**

Risks of being assigned to the surgery group are not greater than the normal risks associated with any orthopaedic operation. These include anaesthetic risks and surgical risks such as nerve/ blood vessel injury including bleeding, wound breakdown, infection, painful hardware irritation requiring removal, elbow stiffness, pain from the fracture not healing, and failure of the implanted hardware including hardware movement.

For those in the no surgery group, risks include pain from the fracture not healing and limitation of elbow movement including weakness. These are risks associated with any patient managed without surgery. If you are in the no surgery group and dissatisfied with your progress at any point, you will be allowed to opt out of the study and undergo delayed surgery.

**7. Will I benefit from the study?**

This study may not directly benefit you as it aims to improve future treatment of patients with similar fractures.

**8. Will taking part in this study cost me anything, and will I be paid?**

There will be no costs to you for participating. You will not be paid for your participation in this study.

**9. How will my confidentiality be protected?**

There will be multiple steps in place to protect your confidentiality. All written documentation will be stored in a study folder. The folder will be kept in a locked filing cabinet at all times. A de-identified copy (only containing a randomly generated 5 digit code at the top) of this data will be faxed to the Principal Investigator’s (Dr John Limbers) private rooms, where it will be stored in a secure filing cabinet. Only research personnel will have access to your information. All participants medical information will be accessed, used, managed and stored in accordance with the NSW Health Records and Information Privacy Act 2002.

**10. What happens with the results?**

We plan to publish the results in a medical journal and present them at scientific meetings. In any publication or presentation, information will be provided in such a way that no participants from the study can be identified. Results of the study will be provided to you, if you wish.

**11. What happens to my treatment when the study is finished?**

Your ongoing treatment will not be affected in any way regardless of whether you decide to participate in the study or not.

**12. What should I do if I want to discuss this study further before I decide?**

When you have read this information, any of the researchers listed above will be happy to answer queries/concerns you may have. If you would like to know more at any stage, please do not hesitate to contact [***Insert site-specific project supervisor’s contact details]***.

**13. Who should I contact if I have concerns about the conduct of this study?**

This study has been approved by the Hunter New England Human Research Ethics Committee (Reference 13/10/16/4.04). Any person with concerns or complaints about the conduct of this study should contact the Research Office who is nominated to receive complaints from research participants. You should contact them on (02) 4921-4950 and quote reference number 13/10/16/4.04

**Thank you for taking the time to consider this study.**

**If you wish to take part in it, please sign the attached consent form.**

**This information sheet is for you to keep.**

**[Insert institutional letterhead]**

**[name of local institution/s where research is being conducted]**

**SOFIE: Surgery for Olecranon Fractures In the Elderly: a randomised controlled trial of operative versus non-operative treatment.**

## CONSENT FORM

1. I ,................................................................................................................. of ................................................................................................................

agree to participate as a subject in the study described in the participant information statement set out above.

2. I acknowledge that I have read the participant information statement, which satisfactorily explains why I have been selected and the aims of the study.

3. Before signing this consent form, I have been given the opportunity of asking any questions relating to any possible physical and mental harm I might suffer as a result of my participation and I have received satisfactory answers.

4. I understand that I can withdraw from the study at any time and all information about me will be destroyed without prejudice to my present or future treatment at **Insert site-specific Hospital**.

5. I agree that research data gathered from the results of the study may be published, provided that I cannot be identified.

6. I understand that if I have any questions relating to my participation in this research, I may contact ***Insert site-specific project supervisor’s contact details***, who will be happy to answer them.

7. I acknowledge receipt of a copy of this Consent Form and the Participant Information Statement.

# Signature of subject Please PRINT name Date

# Signature of investigator Please PRINT name Date

#

# ___________________________________________________________________________
