# Supplementary material for: SOFIE: Surgery for Olecranon Fractures in the Elderly: a randomised controlled trial of operative versus non-operative treatment
Source: BMC Musculoskelet Disord. 2015 Oct 27;16:324. doi: 10.1186/s12891-015-0789-6 (PMC4624605; doi:10.1186/s12891-015-0789-6)

**Patient Data Sheet**

**Name**: _______________________ **DOB** (D/M/Y): __________ **MRN**: _________

**Address**: _________________________________ **Phone**: ________________

**Date of Injury**: _________________ **Date of enrollment**: __________________

**Site of Enrollment: _________________ Surgeon**: _________________

**Side** (circle): Left/ Right **Dominance** (circle): Dominant/ Non-dominant

**Diabetes** (circle): Yes/ No **Smoker** (circle): Yes/ No **Comminution** (circle): Yes/ No

**Study group** (circle): Non-operative/ Operative (Plate/ Tension band wiring)

**PRECEDING PROBLEMS (ONLY FOR INITIAL PRESENTATION)**

Were you having difficulty using the arm (wrist/ elbow/ shoulder) prior to this injury?

Yes/ No

**REVIEW**

**Time** (circle) 3 months/12 months

**Active ROM:**

*Fractured side* Elbow flexion = _____ deg Elbow extension = _____ deg

*Contralateral side* Elbow flexion = _____ deg Elbow extension = _____ deg

**Dynamometer Extension Strength:**

*Fractured side* Strength = ______Nm

*Contralateral side* Strength *= ______*Nm

**Complications since previous review**:

1. Infection (specify treatment if yes): Yes/No
2. Hardware migration: Yes/No
3. Reoperation (if yes, give reason): Yes/No
4. Other (if yes, specify): Yes/No

**DASH: _________**

**Pain VAS: _______**

**EQ VAS: ________**

**Notes**:

**Disabilities of the Arm, Shoulder and Hand (DASH)**

Please rate your ability to do the following activities in the last week by circling the number below the appropriate response.

|  | Activity | No difficulty | Mild difficulty | Moderate difficulty | Severe difficulty | Unable |
| --- | --- | --- | --- | --- | --- | --- |
| 1. | Open a tight or new jar | 1 | 2 | 3 | 4 | 5 |
| 2. | Write | 1 | 2 | 3 | 4 | 5 |
| 3. | Turn a key | 1 | 2 | 3 | 4 | 5 |
| 4. | Prepare a meal | 1 | 2 | 3 | 4 | 5 |
| 5. | Push open a heavy door | 1 | 2 | 3 | 4 | 5 |
| 6. | Place an object on a shelf above your head | 1 | 2 | 3 | 4 | 5 |
| 7. | Do heavy household chores (wash walls, wash floors) | 1 | 2 | 3 | 4 | 5 |
| 8. | Garden or do yard work | 1 | 2 | 3 | 4 | 5 |
| 9. | Make a bed | 1 | 2 | 3 | 4 | 5 |
| 10. | Carry a shopping bag or briefcase | 1 | 2 | 3 | 4 | 5 |
| 11. | Carry a heavy object (over 10 lbs) | 1 | 2 | 3 | 4 | 5 |
| 12. | Change a light bulb overhead | 1 | 2 | 3 | 4 | 5 |
| 13. | Wash or blow dry your hair | 1 | 2 | 3 | 4 | 5 |
| 14. | Wash your back | 1 | 2 | 3 | 4 | 5 |
| 15. | Put on a pullover sweater | 1 | 2 | 3 | 4 | 5 |
| 16. | Use a knife to cut food | 1 | 2 | 3 | 4 | 5 |
| 17. | Recreational activities which require little effort (card playing, knitting) | 1 | 2 | 3 | 4 | 5 |
| 18. | Recreational activities in which you take some force or impact through your arm, shoulder or hand (golf, hammering, tennis) | 1 | 2 | 3 | 4 | 5 |
| 19. | Recreational activities in which you move your arm freely (playing Frisbee, badminton) | 1 | 2 | 3 | 4 | 5 |
| 20. | Manage transportation needs (getting from one place to another) | 1 | 2 | 3 | 4 | 5 |
| 21. | Sexual activities | 1 | 2 | 3 | 4 | 5 |

|  | Activity | Not at all | Slightly | Moderately | Quite a bit | Extremely |
| --- | --- | --- | --- | --- | --- | --- |
| 22. | During the past week, to what extent has your arm, shoulder or hand problem interfered with your normal social activities with family, friends, neighbours or groups? *(circle number)* | 1 | 2 | 3 | 4 | 5 |

|  | Activity | Not at all | Slightly | Moderately | Quite a bit | Extremely |
| --- | --- | --- | --- | --- | --- | --- |
| 23. | During the past week, were you limited in your work or other regular daily activities as a result of your arm, shoulder or hand problem? *(circle number)* | 1 | 2 | 3 | 4 | 5 |

Please rate the severity of the following symptoms in the last week. *(circle number)*

|  | Activity | None | Mild | Moderate | Severe | Extreme |
| --- | --- | --- | --- | --- | --- | --- |
| 24. | Arm, shoulder or hand pain | 1 | 2 | 3 | 4 | 5 |
| 25. | Arm, shoulder or hand pain when you performed any specific activity | 1 | 2 | 3 | 4 | 5 |
| 26. | Tingling (pins and needles) in your arm, shoulder or hand | 1 | 2 | 3 | 4 | 5 |
| 27. | Weakness in your arm, shoulder or hand | 1 | 2 | 3 | 4 | 5 |
| 28. | Stiffness in your arm, shoulder or hand | 1 | 2 | 3 | 4 | 5 |

|  | Activity | No difficulty | Mild difficulty | Moderate difficulty | Severe difficulty | So much difficulty that I can’t sleep |
| --- | --- | --- | --- | --- | --- | --- |
| 29. | During the past week, how much difficulty have you had sleeping because of the pain in your arm, shoulder or hand? *(circle number)* | 1 | 2 | 3 | 4 | 5 |

|  | Activity | Strongly disagree | Disagree | Neither agree nor disagree | Agree | Strongly agree |
| --- | --- | --- | --- | --- | --- | --- |
| 30. | I feel less capable, less confident or less useful because of my arm, shoulder or hand problem? *(circle number)* | 1 | 2 | 3 | 4 | 5 |

**Pain Visual Analog Scale**

Circle the number on the line that matches the pain in your elbow.


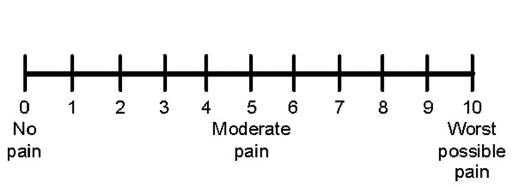


**EQ Visual Analog Scale**


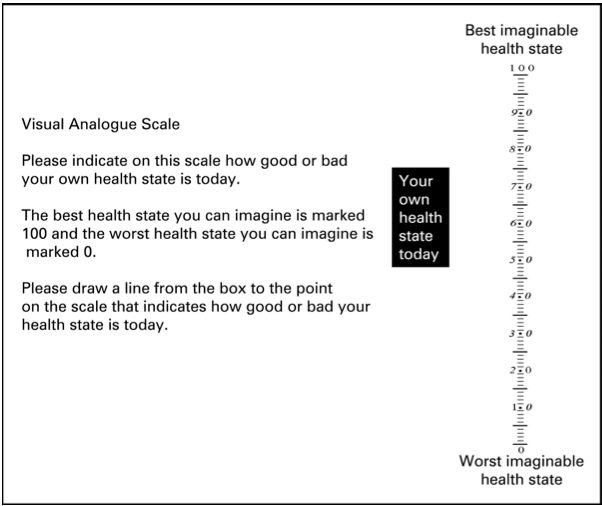

Supplement: Additional file 2: — Patient Information Sheet. (DOCX 90 kb) [file 12891_2015_789_MOESM2_ESM.docx]
